# Energy-Saving Traffic Scheduling in Hybrid Software Defined Wireless Rechargeable Sensor Networks

**DOI:** 10.3390/s17092126

**Published:** 2017-09-15

**Authors:** Yunkai Wei, Xiaohui Ma, Ning Yang, Yijin Chen

**Affiliations:** School of Communication and Information Engineering, University of Electronic Science and Technology of China, Chengdu 610051, China; shaoxingmxh@126.com (X.M.); yn@uestc.edu.cn (N.Y.); yjchen@std.uestc.edu.cn (Y.C.)

**Keywords:** software defined network, software defined wireless rechargeable sensor network, energy saving, traffic scheduling, wireless charger

## Abstract

Software Defined Wireless Rechargeable Sensor Networks (SDWRSNs) are an inexorable trend for Wireless Sensor Networks (WSNs), including Wireless Rechargeable Sensor Network (WRSNs). However, the traditional network devices cannot be completely substituted in the short term. Hybrid SDWRSNs, where software defined devices and traditional devices coexist, will last for a long time. Hybrid SDWRSNs bring new challenges as well as opportunities for energy saving issues, which is still a key problem considering that the wireless chargers are also exhaustible, especially in some rigid environment out of the main supply. Numerous energy saving schemes for WSNs, or even some works for WRSNs, are no longer suitable for the new features of hybrid SDWRSNs. To solve this problem, this paper puts forward an Energy-saving Traffic Scheduling (ETS) algorithm. The ETS algorithm adequately considers the new characters in hybrid SDWRSNs, and takes advantage of the Software Defined Networking (SDN) controller’s direct control ability on SDN nodes and indirect control ability on normal nodes. The simulation results show that, comparing with traditional Minimum Transmission Energy (MTE) protocol, ETS can substantially improve the energy efficiency in hybrid SDWRSNs for up to 20–40% while ensuring feasible data delay.

## 1. Introduction

Wireless Sensor Networks (WSNs) usually suffer from the limitation of energy supply, especially in some rigid application environments, such as hydrological monitoring, disaster warning, mountain battlefield spying, etc. Consequently, the emerging Wireless Rechargeable Sensor Networks (WRSN) [1,2], as a promising technique in the future, have been paid great attention in recent years.

**Figure 1:**
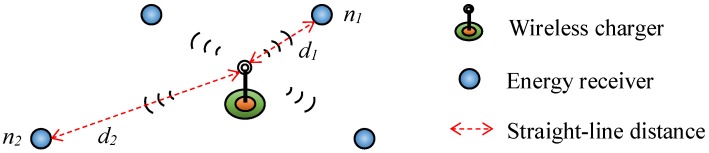
An example of wireless recharging.

However, in many applications, the wireless charger’s energy is also limited. To demonstrate this issue, Figure 1 shows a simple example, which is quite popular in the application of WRSNs. In Figure 1, the energy receivers (i.e., sensor nodes, relay nodes, or sink node), which are numerous in amount but should be cheap in price, are powered by the wireless charger according to their residual capacity. At the same time, the wireless charger’s energy may be supplemented by some energy harvesting method, e.g., solar energy, wind energy, etc., which is restricted by environmental factors and cannot always supply sufficient energy all the time. In such scenarios, the energy saving is still a key problem in WRSNs.

Since the charging efficiency is inversely proportional to the square of distance *d* from the wireless charger to the energy receiver [3], the same amount of energy consumption in different nodes will lead to different burdens on the wireless charger. Investigate nodes $n_{1}$ and $n_{2}$ in Figure 1, which each have a distance of $d_{1}$ and $d_{2}$ from the wireless charger, respectively. When $n_{1}$ consumes 1 unit energy, the wireless charger should provide energy $E_{1}\propto {d_{1}}^{2}$ to supplement the consumption. However, for node $n_{2}$, there is $E_{2}\propto {d_{2}}^{2}$. Supposing that $d_{2}=2d_{1}$, then $E_{2}$ is approximately 4 times of $E_{1}$. Consequently, the energy saving problem in WRSNs is more complicated than normal WSNs. Traditional energy saving schemes in normal WSNs cannot be directly transplanted into WRSNs.

At the same time, the combining of Software Defined Networking (SDN) and WRSNs [4], which is an inexorable trend, brings new opportunities to energy saving issues in WRSNs. SDN can schedule the traffic and energy distribution in the perspective of the whole network, so as to achieve more energy gain while satisfying the service requirements. Nevertheless, the evolution of WRSN to Software Defined WRSN (SDWRSN) is a gradual process. Normal WRSNs and SDWRSNs may exist concurrently in a long time, sometimes even in the same area, for the same set of applications. That is to say, in such networks, sensor nodes with SDN capabilities (SDN nodes) and normal sensor nodes (normal nodes) may coexist. We define such networks as hybrid SDWRSNs. In such hybrid SDWRSNs, the SDN controller can only directly control the SDN nodes, but not the normal nodes, leading to a discount on possible energy saving effect.

Consequently, in the context of hybrid SDWRSNs, the energy saving problem is quite different comparing to normal WSNS, or even normal WRSNs, as follows:

(1) The lifetime of each node is no longer a key problem. Their energy can be supplemented by the wireless charger in time. On the contrary, the total energy consumption of all nodes, or the burden of the wireless charger turns out to be a key problem.
(2) The energy consumption model is different. The geographical distribution of sensor nodes will directly impact the charging efficiency, and thereby change the burden of the wireless charger.
(3) Although the SDN controller can only directly control the SDN nodes to optimize the traffic and energy consumption, this will indirectly influence the inlet flow of normal nodes, and thus change the behaviours of normal nodes indirectly.

Existing studies, which will be demonstrated particularly in Section 2, usually concentrate on several major aspects. For normal WSNs, a great amount of work has been done focusing on how to extend the lifetime for sensor nodes in the energy-constrained network with a special routing algorithm. In WRSNs, most works try to improve energy efficiency by optimizing the deployment or moving route of the wireless charger. A few studies take the energy limitation of wireless charger into account. To the best of our knowledge, no current work has been done focusing on the energy saving issue in hybrid SDWRSNs.

To solve the above problem, in this paper, we put forward an Energy-saving Traffic Scheduling (ETS) algorithm for hybrid SDWRSNs. The ETS algorithm adequately considers the new characters in hybrid SDWRSNs and takes advantage of the SDN controller’s direct control ability on SDN nodes and indirect control ability on normal nodes. The simulation results show that ETS can substantially improve the energy efficiency in hybrid SDWRSNs, and therefore ensure the network working more steadily and chronically. We highlight our contributions of this paper as follows:

Firstly, we derive a mathematical optimization model for the energy efficiency problem in hybrid SDWRSNs. Although it proved to be a non-deterministic polynomial hard (NP-hard) problem, the model provides an outline and paradigm for energy saving problem in hybrid SDWRSNs.

Then, a fast heuristic algorithm known as Energy-saving Traffic Scheduling (ETS) is presented, which takes charging losses and the new energy saving characters into account, and schedules the flows through both SDN nodes (directly) and normal nodes (indirectly).

Finally, we evaluate the performance of the ETS algorithm with simulations. Compared with traditional Minimum Transmission Energy (MTE) protocol, ETS has a much better effect on reducing energy consumption when ensuring feasible data delays.

The remainder of this paper is organized as follows. The related work is reviewed in Section 2. In Section 3, we demonstrate the system model. The mathematical optimization model for hybrid SDWRSN is formulated in Section 4 and the ETS algorithm is described in Section 5. Section 6 displays the simulation results and performance analysis. The conclusions are presented in Section 7 in the end.

## 2. Related Work

To the best of our knowledge, so far there is no work concentrated on energy saving problems in hybrid SDWRSNs. Some related works, which establish a foundation for this problem, can be classified into three categories, as follows.

### 2.1. Energy Saving Studies in WSNs

The energy limitation is always a key problem for various WSNs. A lot of work has been done to reduce energy consumption and prolong the lifetime of WSNs. These studies usually focus on optimizing network deployment [5,6,7,8], power control or sleeping schemes [9,10], ameliorating data processing method [11,12,13], or improving communication protocol [14,15,16,17,18,19]. These studies cannot be directly transplanted into hybrid SDWRSNs, but they provide referable thoughts for the latter.

### 2.2. Energy Harvesting Techniques

At the same time, researchers have explored various methods to acquire sufficient energy for WSNs with the method of energy harvesting. Refs. [20,21] provide an overview on this issue. Instead of equipping an energy harvesting module on every sensor node to gather energy from surrounding energy sources such as solar [22], vibration [23], wind [24], etc., a more economical and controllable way is to use special charging equipment to transmit power to sensor nodes. Magnetic resonance [25], electromagnetic induction [26], laser light sensing [27] and micro-wave conversion [28] are relatively mature technologies in this respect.

These energy harvesting techniques are the basis of WRSNs, and the new charging pattern should be fully considered in the design of energy saving scheme for hybrid SDWRSNs.

### 2.3. Energy Saving in WRSNs

In the wireless rechargeable sensor networks, the energy efficiency issue is still a key problem. In [29], the potential mobility of sensor nodes has been exploited and a new metric Quality of Energy Provisioning (QoEP) is proposed, so as to characterize the expected portion of time that a mobile node can sustain normal operation in WRSNs, and to evaluate the impact of mobility on energy provisioning in WRSNs. Considering that some wireless chargers are not fixed, there are some studies based on the movable charger (MC), such as [30,31,32,33]. In [30], Mo et al. study addressing the MC scheduling, the moving and charging time allocation simultaneously to save total energy and formulate this problem as a mixed integer linear program, which is solved by a novel decentralized method based on Benders decomposition. Toyoda considers the situation that the MC can collect packets as well as the static sink does in [31], and proposes a closer destination selection scheme, which improves the packet arrival rate and energy efficiency compared to the conventional scheme. As for [32], the researchers pay attention to large-scale WRSNs with multiple MCs and develop a heuristic algorithm to find the minimum number of MCs for the sensor nodes to work continuously. Hu and Wang [33] consider the scheduling of the charging mission to ensure that all sensor nodes will be working within a target lifetime.

The sensor nodes and energy charger may be fixed in preset locations. In this situation, existing methods to improve the energy efficiency are mainly by optimizing the deployment of chargers. In [34], researchers present two greedy algorithms to deploy as few as possible chargers. In [35], the Particle Swarm Charger Deployment (PSCD) algorithm using the Particle Swarm Optimization (PSO) concept is proposed to optimize WRSN charger deployment. A layoff algorithm to combine simulated annealing-based algorithm in [36] is discussed to work on this issue. In addition, some power control and protocol joint design are also considered, e.g., [37,38]. Pal and Nasipuri [37] consider reducing overhearing by reducing the neighborhood size using transmission power control as well as route adaptations, and implements a cooperative joint Power COntrol and Routing (PCOR) scheme for rechargeable sensor networks. Liu et al. [38] put forward a local algorithm, called SnapIt, to adapt the sampling rate with the objective of maintaining the battery at a target level.

However, all the above studies on energy saving in WRSNs try to save energy through optimized deployment of wireless chargers. The energy saving scheme of traffic scheduling is still a blank for WRSNs.

In summary, current studies on energy saving for WSNs or WRSNs are not suitable for hybrid SDWRSNs. New schemes are urgent to be developed to satisfy the energy saving requirement in hybrid SDWRSNs.

## 3. System Model

**Figure 2:**
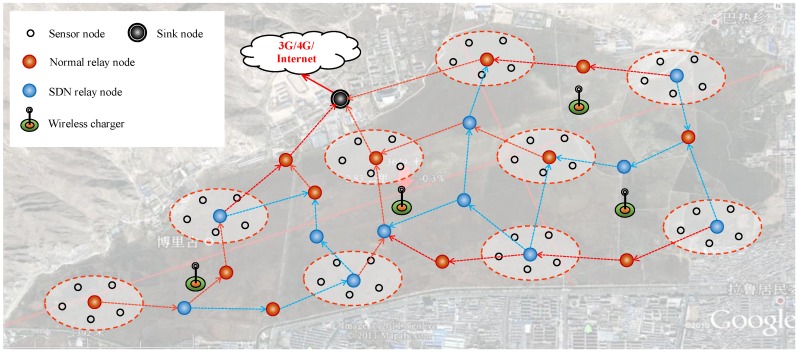
System model.

As shown in Figure 2, this paper considers a typical kind of hybrid SDWRSN, where four kinds of nodes are included: sensor nodes, relay nodes, a sink node and some wireless chargers. The sensor nodes collect required information according to some predefined period or discipline, and transmit them to the sink node via some relay nodes. The sensor nodes are dispersedly deployed over the target monitoring area. A certain amount of sensor nodes close to each other form a cluster. Each cluster has a cluster head, which can be dynamically selected by currently existing schemes. The cluster heads can relay data from the cluster it belongs to, as well as data from another cluster head. Therefore, in this paper, we also refer to cluster head as relay node for simplicity.

Basically, there are two kinds of relay nodes: normal relay node (or shorted as normal node) and SDN relay node (or shorted as SDN node). The normal nodes run traditional routing protocols, such as MTE, Ad hoc On-demand Distance Vector Routing (AODV), etc. In this paper, we use MTE as routing protocol among normal nodes, since MTE has good performance in reducing the energy consumption of the WSN nodes. As to the SDN nodes, a common SDN controller can calculate the optimal flow table according to the whole network’s topology and flow distribution. The controller can talk to the SDN nodes via 3G/4G (when a cellular network is available) or some other long distance communication methods such as LoRa (3 km urban, 30 km rural). This will not add obvious economic burden since only a part of relay nodes or sensor headers are SDN nodes. In addition, this will not introduce obvious additional energy consumption in the pattern discussed in this paper, as follows: (1) When the locations of the network nodes are static, the network topology is relatively stable. At the same time, the flow characters are usually steady since the services in WSN are generally periodical. (2) The update of the flow table may occur in two patterns: (a) SDN controller can update the flow table periodically. The period can be relatively long, for example, 10 min one time, 1 h one time or even several hours one time; (b) triggered by an event, such as node joining/existing, etc. Therefore, the low frequency update and occasionally triggered update will not introduce obvious additional energy consumption.

At the same time, some wireless chargers are deployed in proper locations to replenish the energy consumption of network devices, i.e., sensor nodes, the relay nodes, and the sink node. Consequently, considering that the sensor nodes, the sink node, and the SDN controller work according to their predefined pattern and will not be influenced by the algorithm proposed in this paper, we need not count the energy consumption of these three parts, and concentrate on the algorithm’s effect on the relay nodes.

## 4. Mathematical Optimization Model

In this section, we firstly derive the node energy consumption model, and then present the mathematical optimization model of the traffic scheduling problem in hybrid SDWRSNs.

### 4.1. Node Energy Consumption

For a certain relay node *v* that receives data from some node *u* and transmits data to some other node *w* in a time span *t*, there are four states: transmitting state, receiving state, idle state and sleeping state. We discuss below the energy consumption of these four states, respectively, referring to the energy consumption model in [39].

(1) Transmitting state. When node *v* is sending $k_{vw}$ bits data to a random node *w*, the energy consumption $E_{Tvw}$ mainly relates to the amount of transmitted data $k_{vw}$ and the distance to the receiver node *w*, which is denoted with $d_{vw}$. $E_{Tvw}$ composes of two parts. The first part is the energy to run radio electronics $E_{Tvw-elec}=E_{elec}\times k_{vw}$. $E_{elec}$ depends on factors such as the digital coding, modulation, filtering, and spreading of the signal. Another part is $E_{Tvw-amp}$, the energy consumed to amplify the signal. $E_{Tvw-amp}$ depends on the distance $d_{vw}$ from node *v* to target *w*, and there is a threshold $d_{0}$. When $d_{vw}<d_{0}$, the free space model $\epsilon_{fs}$ is used. Otherwise, the multipath model $\epsilon_{mp}$ is used, i.e.,

(1) $$E_{Tvw-amp}=\left\{\begin{array}{cc} k\times \epsilon_{fs}\times d_{vw}^{2} & d_{vw}<d_{0},\\ k\times \epsilon_{mp}\times d_{vw}^{4} & d_{vw}\geq d_{0}. \end{array}\right.$$

Therefore, the energy consumption in transmitting state $E_{Tvw}=E_{Tvw-elec}+E_{Tvw-amp}$.

(2) Receiving state. In this state, node *v* is receiving some $k_{uv}$ bits data from a random node *u*. The energy consumption $E_{Ruv}$ for node *v* only concerns about the radio electronics, i.e.,

(2) $$E_{Ruv}=E_{Ruv-elec}=E_{elec}\times k_{uv}.$$

(3) Idle state. In this state, the only function for a node is to keep listening to the channel and being ready to receive data. Generally, the idle power consumption $P_{Iv}$ is 0.8–1 times of receiving power $P_{Rv}$. For simplicity and without loss of generality, we set $P_{Iv}=P_{Rv}$, that is:

(3) $$P_{Iv}=P_{Rv}=\frac{E_{Ruv}}{t_{Ruv}}=\frac{E_{elec}\times k_{uv}}{t_{Ruv}}=E_{elec}\times R,$$

where $t_{Ruv}$ is the time used for node *u* to transmit $k_{uv}$ bits data to node *v*, and *R* is the data rate of the wireless link from node *u* to node *v*. Thus, the energy consumption $E_{Iv}$ of an idle node *v* can be calculated as:

(4) $$E_{Iv}=P_{Iv}\times t_{Iv},$$

where $t_{Iv}$ is the time of node *v* in idle state.

(4) Sleeping state. In this state, the energy consumption $E_{Sv}$ is very little and negligible. A common energy-saving mechanism is to allow the node to sleep when there is no task interested and wakes it up when communication happens.

According to the analysis above, for a relay node *v*, the energy consumption in a time span *t* is:

(5) $$E_{v}=\sum_{w\in \{\mathrm{neighbours}\;\mathrm{of}\;v\}}E_{Tvw}+\sum_{u\in \{\mathrm{neighbours}\;\mathrm{of}\;v\}}E_{Ruv}+E_{Iv}+E_{Sv}.$$

However, in WRSN, what we are really concerned about is the actual energy $E_{cv}$ provided by a wireless charger *c* to node *v*. When radio waves travel in space, their powers attenuate with increased travel distance. The wireless charger usually charges the devices that are not far from itself. Long distance or obstructions may greatly recede the charging efficiency, which can be avoided by deploying more wireless chargers in practice. Consequently, we can adopt free space propagation model. According to Friis’s free space equation, the receive power $p_{r}$ of RF signal can be expressed as

(6) $$p_{r}=G_{s}G_{r}{\left(\frac{\lambda }{4\pi d}\right)}^{2}p_{0},$$

where $G_{s}$ is the source antenna gain, $G_{r}$ is the receive antenna gain, λ is the wavelength, *d* is the distance between the source node and the receiver node, and $p_{0}$ denotes the transmitting power of the source node [3].

Assume the maximum battery volume of node *v* is $E_{mb-v}$, and the battery is full at the starting time. After a time span *t*, the rest energy in node *v*’s battery is $E_{rest-v}$. Let $d_{cv}$ denote the distance from node *v* to its responsible wireless charger *c*, $P_{cv}$ denote the charging power from the wireless charger *c* to node *v*, and $t_{cv}$ denote the total charging time of node *v*. Considering $d_{cv}$ should be less than $d_{0}$ to ensure feasible charging efficiency (Equation (1)), there is:

(7) $$\frac{P_{cv}t_{cv}}{A\times d_{cv}^{2}}=E_{v}+E_{rest-v}-E_{mb-v},$$

where *A* is a constant coefficient. Note that, in Equation (7), the charging time $t_{cv}$ may be accumulated from many discrete time slots in time span *t*. When t→∞, there should also be $t_{cv}\to \infty$. In this case, we have:

(8) $$\frac{P_{cv}}{A\times d_{cv}^{2}}=\lim_{t_{cv}\to \infty }\left\{\frac{E_{v}}{t_{cv}}+\frac{E_{rest-v}}{t_{cv}}-\frac{E_{mb-v}}{t_{cv}}\right\}\overset{\left(a\right)}{=}\frac{E_{v}}{t_{cv}},$$

where *(a)* follows that $E_{rest-v}$ and $E_{mb-v}$ are both limited constants, and $E_{v}$ is a function of *t* ($t\geq t_{cv}$). Therefore, when t→∞, we can consider the energy consumption $E_{v}$ equals to the energy charged to node *v*. That is to say, when t→∞,

(9) $$E_{v}=\frac{P_{cv}t_{cv}}{A\times d_{cv}^{2}}.$$

Suppose when a node *v* consumes $E_{v}$ energy, the responsible wireless charger *c* should consume $E_{cv}$ energy to compensate the energy consumption (i.e., $E_{v}$ consumption at node *v*). Combining with Equation (9), there is:

(10) $$E_{cv}=P_{cv}t_{cv}=A\times E_{v}\times d_{cv}^{2}.$$

### 4.2. Mathematical Optimization Model

In order to get mathematical optimization model of the traffic scheduling problem in hybrid SDWRSNs, we assume *V* is the set of relay nodes (including sink node) in the network and *C* is the set of wireless chargers. *N* and *D*, respectively, represent the subset of normal nodes and the subset of the SDN nodes. The sink node is expressed as *s*. Obviously, there is N⊆V, D⊆V, and N+D+{s}=V. The optimization objective is:

(11) $$\min:\sum_{v\in V,\;v\neq s,\;c\in C}E_{cv}.$$

For this problem, there are some constraint conditions to be followed. The flow conservation conditions are first. For node *v*, in a given time span *t*, the data it transmitted ($k_{Tv}$) equals to the data it received from the sensor subnet ($k_{v}$) and other relay nodes ($k_{Rv}$), except the sink node which only receives data from other nodes. For the entire network, the amount of data received is equal to the amount of data transmitted. Then, the constraints come as shown in Equations (12)–(14):

(12) $$k_{Tv}=k_{v}+k_{Rv},\;\forall v\in V,v\neq s,$$

(13) $$k_{Rs}=\sum_{\forall v\in V,\;v\neq s}k_{v},$$

(14) $$\sum_{\forall v\in V}k_{Tv}=\sum_{\forall v\in V}k_{Rv}.$$

Another basic constraint is the capacity. Practically, the real data transmission rate is smaller than the nominal data rate. It is a general rule in communications to set a traffic allocation upper bound, which is lower than the channel’s nominal data rate. Denote the data rate with *R* and the threshold with β, there is:

(15) $$\frac{k_{Tv}+k_{Rv}}{t}\leq \beta R,\;\forall v\in V,\;0<\beta \leq 1.$$

For the normal nodes that run MTE protocol, the routes they choose have the minimum transmission energy. Assume $r_{vw}$ is the minimum transmission energy from node *v* to node *w*, and use a binary variable $u_{vlw}$ to show whether the node *l* (which is one of the one-hop neighbours of node *v*) is on the way from node *v* to node *w* and is equal to 1 when it is on that way. Assuming *M* is an arbitrarily large value and *m* is an arbitrarily small value, then there are two constraints as below:

(16) $$\begin{array}{cc} r_{vl}+r_{lw}-r_{vw}\leq \left(1-u_{vlw}\right)M & \forall v,l,w\in N,\\ r_{vl}+r_{lw}-r_{vw}\geq \left(1-u_{vlw}\right)m & \forall v,l,w\in N, \end{array}$$

when a node *l* is not on the way from node *v* to node *w*; on node *l*, there should be no data sent from node *v* to node *w*. When $k_{vlw}$ represents the amount of data transferred from node *v* via node *l* to node *w*, then:

(17) $$k_{vlw}\leq u_{vlw}\times k_{lw}\;\forall v,l,w\in N.$$

For MTE, the route for each node is unique, therefore,

(18) $$\sum_{l}u_{vlw}=1,\;\forall v,l,w\in N.$$

From Equation (7), to a node *v*, if the energy consumption speed is larger than the energy charging speed, the node will eventually drain out its power and become inactive. To avoid this problem, at any time there should be

(19) $$E_{rest-v}=E_{mb-v}+\frac{P_{cv}t_{cv}}{A\times d_{cv}^{2}}-E_{v}>0.$$

Note that, in a short time span, i.e., from the starting time to any time point *t* (t<<∞), there may exist $\left(P_{cv}t_{cv}\right)/\left(A\times d_{cv}^{2}\right)<E_{v}$ since the battery energy can make up for the difference. However, when $t_{2}-t_{1}\to \infty$, there is $\left(P_{cv}t_{cv}\right)/\left(A\times d_{cv}^{2}\right)=E_{v}$, which is consistent with Equation (10). When $E_{rest-v}$ keeps decreasing and tends to 0, the charging power $P_{cv}$ should be increased, or the charging time $t_{cv}$ should be lengthened, so as to ensure constraint Equation (19) always holds.

At the same time, increasing the charging power or charging time will bring a heavier burden to the wireless chargers. Although we can set the chargers with appropriate energy harvesting capability in network designing phase, or deploy them at more optimized locations, there is an inevitable problem that the energy harvesting efficiency of the wireless chargers may be lower than the total energy consumption speed of their subordinative nodes, especially when the efficiency decreases because of devices ageing, environmental influence, or network nodes increasing, etc. This is just the problem that we want to solve in this paper, which tries to decrease the total energy burden of the wireless chargers, just as depicted in Section 1 and the optimization objective.

In literature [40], Fortz and Thorup propose an optimization program to minimize the total communication cost of a given network, subject to the constraints of channel load, traffic demands and channel costs. Investigating our optimization model and the model proposed in [40], we can find the constraints of the programming considered in the latter from a subset of the constraints in the former. Since it has proved to be an NP-hard problem in [40], the model in this paper is also a NP-hard problem. Nevertheless, when a similar SDWRSN network is planned to be deployed, and some parameters have been determined such as the packet size, data period, etc., we can put known parameters into the model and adjust unknown parameters to estimate the lower bound of possible energy consumption or some other metrics. This can be helpful in optimizing the power of each wireless charger, the density of the sensor nodes, the node allocation, and so on. In what follows, we propose a fast heuristic algorithm to decrease the energy consumption in hybrid SDWRSNs.

## 5. Energy-Aware Traffic Scheduling Algorithm

As mentioned above, the problem of energy-saving traffic scheduling in hybrid SDWRSNs is NP-hard. To prolong the steadily working time of the networks under new environments, we should fully consider the new characters in hybrid SDWRSNs, and take advantage of the SDN controller’s direct control ability on SDN nodes and indirect control ability on normal nodes. Consequently, based on the above idea as well as the fast heuristic method, in this paper, we propose an Energy-aware Traffic Scheduling (ETS) Algorithm for hybrid SDWRSNs. The ETS Algorithm improves the energy efficiency by more energy efficient traffic scheduling schemes, which mainly solves the following three problems:

(1) Enlarging the amount of sleeping nodes while satisfying the service requirements. Different from traditional energy saving algorithms in normal wireless sensor networks, in the effective charging scope of wireless chargers, ETS does not need to consider energy consumption balance among different nodes, or single node’s lifetime. ETS algorithm converts the energy consumption of each node into the final energy consumption of the wireless charger, and pays attention to minimize the finally total energy consumption.
(2) The trade-off among different nodes that would be turned into sleeping state. As described above, the same energy consumption in different nodes will result in a different burden on the whole network, or specifically, to the wireless chargers. The ETS algorithm prefers to turn nodes farther to wireless chargers into sleep so as to decrease the energy loss in charging.
(3) The indirect control ability of SDN controllers on normal nodes. In the process of the ETS algorithm, the SDN controller selects the directions of flows on SDN nodes and thus influences the inputted flows on normal nodes, so as to change the normal nodes on the selected path, and achieve a more energy efficient traffic scheduling pattern.

In this paper, while the SDN nodes choose routes according to the flow table determined by the SDN controller, the normal nodes are running MTE protocol. For convenience, the route calculated according to MTE protocol are called MTE route in this paper. When an MTE route of a certain flow has at least one SDN node, we define this flow as an SDN flow; otherwise, it is a normal flow.

Algorithm 1 describes the process framework of ETS. According to current flow distribution and network topology, we choose an MTE route for each flow and judge whether it is an SDN flow or a normal flow. For the normal flows, the SDN controller cannot schedule them directly. Therefore, the nodes on the routes for normal flows must be active. The normal flows should be distributed according to their MTE routes in the first place. Then, for the SDN flows, the first SDN node on its MTE route should be recorded. Algorithms 2 and 3 embody the advantages of SDN nodes provided as compared to the normal nodes, i.e., on the basis of MTE, adjusting the distribution of flows after they arrive the SDN nodes. Algorithm 2 is used to find all the optional paths (as defined in Definition 1) for an SDN flow. In order to take the energy consumed by wireless chargers into account, the actual transmitting energy $E_{c-Tuv}$ should be calculated with the formula below:

(20) $$E_{c-Tuv}=A\times \left(E_{Tuv}\times d_{cv}^{2}+E_{Ruv}\times d_{c^{\prime }u}^{2}\right)\;c,c^{\prime }\in C.$$

**Algorithm 1** Energy-Saving Traffic Scheduling**Input:** The set of nodes *V*, the set of wireless chargers *C*, the set of flows *F***Output:** The set of nodes in working state *A*1:Initialize: A←Ø;2:Find the MTE routes for each f∈F;3:**for** each f∈F **do**4:  **if** *f* is an SDN flow **then**5:   A←A+n, where *n* is the nodes on the MTE route from the source node to the first SDN node in flow *f*;6:   Run *optional paths search function* to find the optional paths $P_{f}$ for flow *f*;7:  **else**8:   A←A+n, where *n* is the nodes on the MTE route in flow *f*;9:   F←F-f;10:  **end if**11:**end for**12:**while** F≠Ø **do**13:  **for** each f∈F **do**14:   **if** $|P_{f}|=1$ **then**15:    A←A+n, where n is the nodes on the unique optional route of flow *f*;16:    F←F-f;17:   **end if**18:  **end for**19:  Run *optional path selection function* to find an optional path $P_{f}$ for the corresponding flow *f*;20:  A←A+n, where n is the nodes on $P_{f}$;21:  Update the size of flow *f*;22:  **if** the size of flow *f* equals to 0 **then**23:   F←F-f;24:  **end if**25:**end while**26:**return** *A*.

When the amount of SDN nodes increases, there may be multiple SDN nodes in an optional path. If we search the optional paths on every SDN node in the path, it will be considerably complicated to find all the optional paths with a high computational complexity. Consequently, to simplify the calculation, except the first SDN node, the rest SDN nodes on this route do not try to optimize the route again. Therefore, the optional paths for an SDN flow is the acyclic MTE routes from the direct neighbours of the first SDN node to the destination.

**Algorithm 2** Optional Paths Search Function**Input:** A flow *f*, the first SDN node *s* on the MTE route of *f*, the set of neighbours of *s*$V_{s}$**Output:** The set of optional paths $P_{f}$1:**for** each $v\in V_{s}$ **do**2:  Find the MTE route $p_{v}$ from node *v* to the destination of flow *f* with the actual energy consumption calculated by formula (20);3:  $p_{v}=p_{v}+s$;4:  Delete the loop in $p_{v}$;5:  $P_{f}\leftarrow P_{f}+p_{v}$;6:**end for**7:**return** $P_{f}$.

**Definition** **1** (Optional paths)**.** The optional paths are specifically defined for an SDN flow. When an SDN flow arrives the first SDN node on its MTE route, the optional paths are all the possible routes from this SDN node to the sink node. The next hop of an SDN node on the optional path can be any neighbour of it, and the next hop of a normal node is chosen according to MTE.

Each SDN flow has one or more optional paths. For the SDN flows with only one optional path, ETS can directly allocate these flows on their unique optional paths. The nodes on the optional paths should also be in a working state. For the SDN flows with multiple optional paths, Algorithm 3 is designed for them to select an optimal path. Some related parameters are as follows: 

count: the amount of nodes that are currently in sleeping state in a possibly used optional path.

*r*: the largest amount of flows that may go through the node. If a node is on the optional path for a flow, then r=r+1.

$\bar{r}$: the average amount of flows that may go through the nodes on the optional path, and $\bar{r}=\frac{\left(\sum r\right)}{n}$, where *n* is the number of nodes on the optional path.

cap: the channel capacity for an optional path can be used, which is equal to the minimum channel capacity of the channels on the path.

restsize: the rest channel capacity for an optional path can be used after allocating the flow. restsize=cap-flowsize. When restsize≥0, it means that the corresponding flow can be totally allocated on this path; otherwise, it means that only a part of the flow can be allocated on this path.

In Algorithm 3, we choose one optimal path from the optional paths according to the following sequences: (1) We preferentially choose the path with the minimum count, which obviously allows more nodes to be turned into sleeping state. (2) If there are several paths having the same minimum count, we arrange these paths in descending order according to $\bar{r}$ and restsize and choose the first path with a positive restsize. When some optional paths have the same count, the optional path with the maximum $\bar{r}$ may have a negative restsize, which means that some parts of the path cannot transmit more data. At the same time, the $\bar{r}$ of this optional path is high and then the amount of flows that are influenced is relevantly large. Consequently, there are more flows that should be allocated on the paths with a higher count, and this is not good to save energy. Choosing the first path with a positive restsize can not only guarantee the path has a $\bar{r}$ as large as possible, which means that the path may have a higher possibility to accommodate more flows, but also make sure the nodes on the path could be used in the following allocation. (3) If all optional paths have a negative restsize, we choose a path with the minimum $\bar{r}$ so as to affect less paths. According to the rules above, we select an optimal path and allocate the corresponding flow. The nodes in the selected path should be on working state. The previously selected paths will influence the selection in the later. After allocating a flow on a selected path, the count, $\bar{r}$ and restsize for other optional paths should be recalculated, until each flow is allocated with a proper path.

**Algorithm 3** Optional Path Selection Function**Input:** The set of optional paths $P_{f}$ of all flows *F***Output:** A selected optional path *p* and its corresponding flow *f*1:**for** each p∈P **do**2:  Calculate the count, restsize, and cap of *p*;3:**end for**4:Arrange *P* in ascending order according to count;5:Initialize i←0, a temporary set of optional paths $P_{t}\leftarrow Ø$;6:**for** i=0;i≤|P|;i++ **do**7:  **if** count[i]==count[0] **then**8:   $P_{t}\leftarrow P_{t}+P\left[i\right]$;9:  **end if**10:**end for**11:Arrange $P_{t}$ in descending order according to $\bar{r}$ and restsize;12:**for** $i=0;\;i\leq |P_{t}|;\;i++$ **do**13:  **if** restsize[i]>0 **then**14:   break;15:  **end if**16:**end for**17:**return**p←P[i] and the corresponding flow *f*.

## 6. Simulations and Results

**Figure 3:**
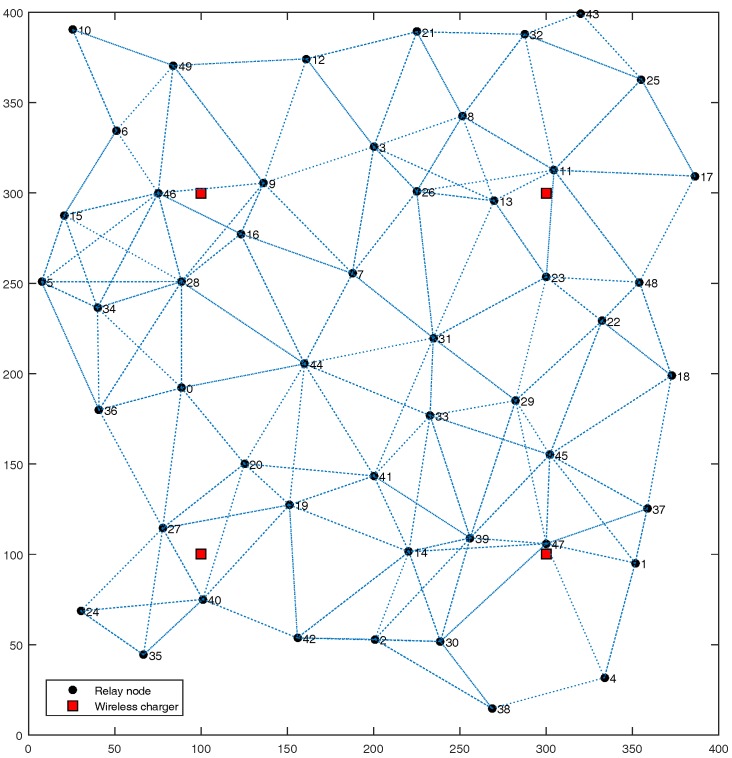
Simulation network topology.

In this section, we evaluate the performance of ETS algorithm via simulations implemented by C++. To ensure the generality of the simulation results, we randomly generate a network as shown in Figure 3, and set the network parameters according to literature [39], as depicted in Table 1. The network includes 50 relay nodes randomly distributed, nearly half of them are selected as cluster heads of the corresponding clusters, which are not pictured out for clearness in Figure 3. Four wireless chargers are deployed uniformity in the target area, which are represented by red squares. Two nodes that can directly communicate with a wireless channel are linked with an imaginary line. Like many works such as [39,41], a simplified model is considered in this paper for communications energy consumption in consideration of path losses. Both the free space ($d^{2}$ power loss) and multipath fading ($d^{4}$ power loss) channel models are employed, depending on the distance between the transmitter and receiver. Power control can be used to compensate for this loss. If the distance is less than a threshold $d_{0}$, the free space model is used; otherwise, the multipath model is adopted. The performance is compared with the MTE algorithm, which has been enhanced for hybrid SDWRSNs.

**Figure 4:**
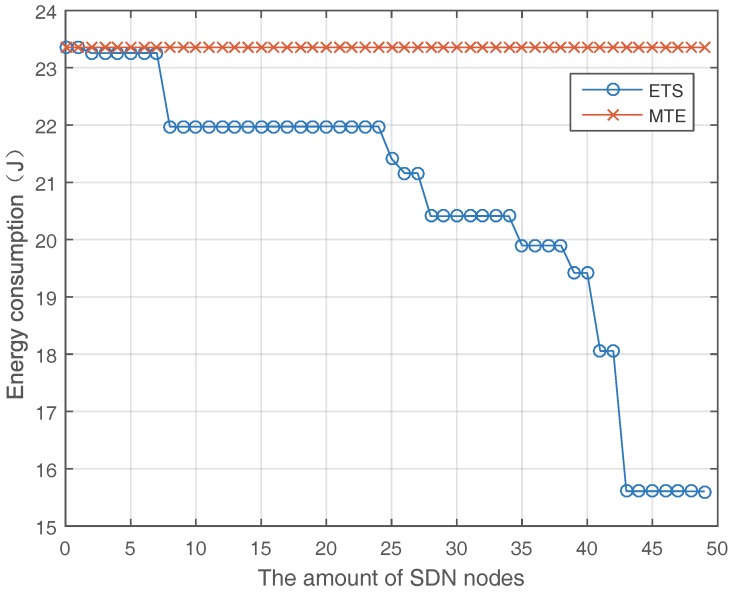
Energy consumption with different SDN nodes.

**Figure 5:**
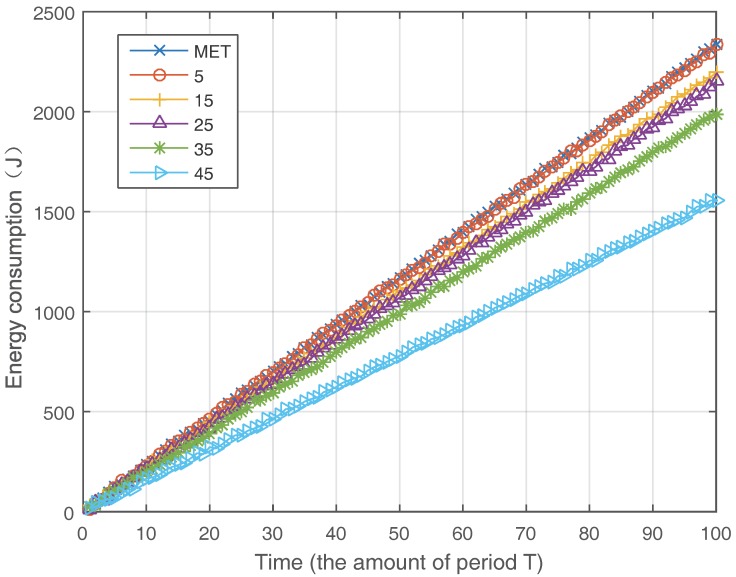
Energy consumption increasing with time.

Figure 4 shows the tendency of energy consumption when the amount of SDN nodes increases. Obviously, the energy consumption of ETS algorithm declines with the increasing of SDN nodes and improve the energy efficiency for up to 20–40% comparing with MTE algorithm. This is because that more SDN nodes in the network can provide higher traffic scheduling ability to save more energy. Investigating Figure 4, we can find sometimes that the amount of increase of SDN nodes does not directly lead to reduction in energy consumption, e.g., from eight SDN nodes to 24 SDN nodes. There are three reasons for this phenomenon. Firstly, the new SDN node may not be the first SDN node on the MTE route of a flow and the ETS algorithm only schedules the flow at the first SDN node. Secondly, the new SDN node is the first SDN node on an MTE route, but there is only one optional path, and then there is no influence on the result. Thirdly, the new SDN node is the first SDN node and there are multiple optional paths, but the optional path chosen is the same as MTE route, which results in the same energy consumption as before. To show this tendency more clearly, we demonstrate the energy consumption in 100 periods when different amounts of SDN nodes are employed in Figure 5.

**Figure 6:**
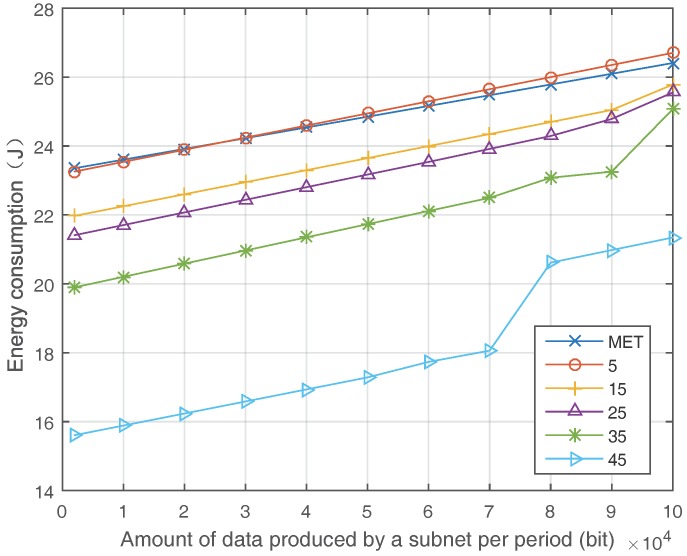
Energy consumption with different load.

Figure 6 shows the influence of network load on the energy consumption. We measure the energy consumption with the increasing of the amount of data produced by a subnet (or cluster) per period. The comparison is implemented among MTE and ETS with a different amount of SDN nodes. In the network with five SDN nodes, the energy consumption of ETS is slightly higher than that of MTE. This is because ETS will alleviate the load of wireless links that exceed the threshold β, but MTE does not. Then, when the amount of SDN nodes grows, the ETS algorithm shows an increasingly larger advantage compared with the MTE algorithm.

**Figure 7:**
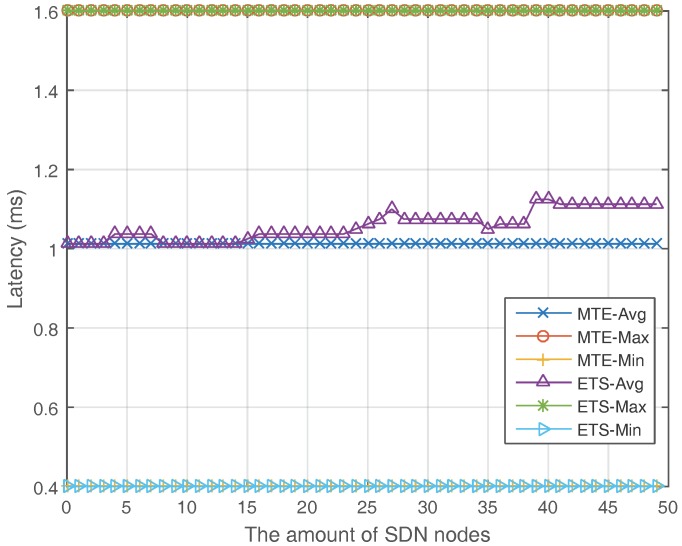
Latency with a different amount of SDN nodes.

**Figure 8:**
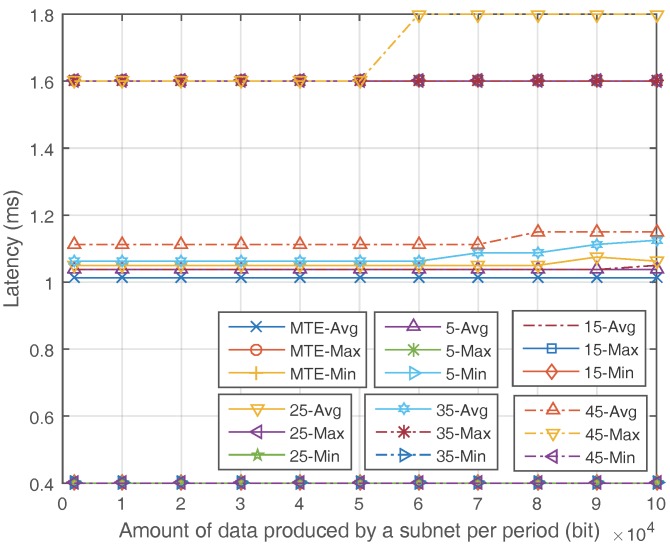
Latency with different network data load.

Figure 7 and Figure 8 show the latency of the MTE algorithm and ETS algorithm. In Figure 7, the minimum and maximum latency of ETS are the same as MTE when the amount of SDN nodes grows. The average latency of ETS is slightly higher than MTE when the ratio of SDN nodes is high because the flows may pass a little longer route when they are converged to save energy. Figure 8 shows the relationship between latency and the network load. For ETS, the average latency remains steady when the network load is under a certain threshold, and then grows slightly. In summary, the ETS algorithm do have a little longer average latency compared to the MTE algorithm. However, the increase in latency is so few that it will not influence the quality of service and can be omitted.

**Figure 9:**
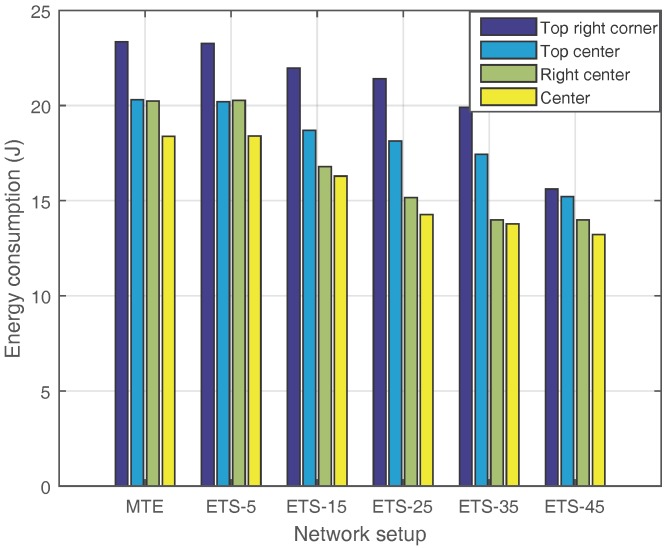
Energy consumption of different sink node location.

Figure 9 shows the impact of the sink node’s position on the energy consumption. We choose four locations for comparison: top right corner, top center, right corner and center in the network. The MTE algorithm and ETS algorithm with different SDN nodes are all compared. The simulation results show that the location of the sink has a direct impact on the energy saving effect, raising the demand on the optimization of sink node deployment. Considering that this issue has been fully investigated in various WSNs, we can directly adopt current solutions when the ETS algorithm is implemented.

Consequently, the ETS algorithm can achieve obvious energy efficiency compared with the traditional MTE algorithm while ensuring feasible data delay.

## 7. Conclusions

A mathematical optimization model for the energy efficiency problem in hybrid SDWRSNs is derived, which provides an outline and paradigm for energy saving problem in hybrid SDWRSNs. Then, we put forward an Energy-saving Traffic Scheduling (ETS) algorithm for hybrid SDWRSNs. The ETS algorithm takes charging losses and the new energy efficiency requirements into account, and schedules the flows through both SDN nodes (directly) and normal nodes (indirectly). The simulation results show that ETS can substantially improve the energy efficiency in hybrid SDWRSNs for up to 20–40% compared with traditional routes with feasible data delay. Therefore, ETS can make the network working more steadily and effectively for a much longer term.

In this paper, we only use the first SDN node along a source-destination pair, so as to avoid too high algorithm complexity. In future work, some improved algorithm that can employ all SDN nodes along a source-destination pair with low complexity can be studied, which might further improve the energy efficiency while ensuring feasible data delay. 

## Figures and Tables

**Table 1:** The simulation parameters.

Parameter	Value
Network area	400 m × 400 m
Number of relay nodes	50
Period	1 s
Data rate	1,000,000 bits/s
Bit error rate	From $1\times 10^{-5}$ to $1\times 10^{-4}$, normally distributed
$E_{elec}$	50 nJ/bit
$\epsilon_{fs}$	10 pJ/bit/m${}^{2}$
$P_{I}v$	$5\times 10^{7}$ nJ/s
Positions of wirelss chargers	(100,100),(100,300),(300,100),(300,300)
